# Functional Biomechanical Analysis of Javelin Throw Technique in Junior Athletes

**DOI:** 10.3390/jfmk11020145

**Published:** 2026-03-31

**Authors:** Ligia Cuciorovschi, Denisa-Iulia Brus, Ștefan Teriș, Răzvan Sandu Enoiu

**Affiliations:** 1 Interdisciplinary Doctoral School, Transilvania University of Brașov, 500036 Brașov, Romania; ligia.cuciorovschi@unitbv.ro; 2 Department of Motor Performance, Transilvania University of Brașov, 500036 Brașov, Romania; stefan.teris@unitbv.ro (Ș.T.); razvan.enoiu@unitbv.ro (R.S.E.)

**Keywords:** functional biomechanics, javelin throw, sport technique, movement analysis, junior athletes, performance

## Abstract

**Background:** Javelin throw performance is strongly influenced by the coordination of the kinetic chain and by key biomechanical parameters related to technique execution. Understanding the functional biomechanical characteristics of javelin throw technique in junior athletes is essential for optimizing performance. **Methods:** This study investigated the biomechanical characteristics of javelin throw technique in junior athletes using an applied motion analysis approach. Kinematic, spatiotemporal, and performance-related variables were assessed during throwing trials at two evaluation time points, before and after a structured biomechanics-informed training period, using motion analysis tools and wearable measurement systems as instruments. **Results:** Significant pre–post changes were observed in technique-related variables (release angle and coordination indices) and in performance outcomes (throwing distance and ball throw speed). **Conclusions:** These findings highlight the functional relationships between biomechanical technique variables and javelin throw outcomes in junior athletes and suggest that field-based biomechanical monitoring can be useful for tracking technique-related changes during structured training in junior throwers.

## 1. Introduction

Sports training is a dynamic and interdisciplinary process integrating biomechanics, physiology, and motor control to optimize athletic performance. Within this framework, training is conceptualized as a regulated process aimed at achieving specific performance objectives through the coordinated interaction of the athlete, coach, training content, and control mechanisms applied during both preparation and competition [1,2,3]. The continuous adaptation of training components is required to ensure effective transfer to competition [4,5].

In contemporary sport, technological and methodological advancements have substantially influenced athlete preparation by enabling objective assessment of movement mechanics and performance determinants [6,7]. The integration of motion analysis systems, inertial measurement tools, and video-based kinematic analysis has enhanced the capacity to evaluate biomechanical variables in field settings. Wearable inertial sensors have demonstrated acceptable validity and reliability for joint kinematics and dynamic sport-specific movements, including throwing and jumping tasks, although limitations related to soft-tissue artifact and integration drift must be considered [8,9]. Such technologies enable practical biomechanical monitoring in ecologically valid training environments. Athletic performance depends on the interaction of biomotor capacities, particularly strength and speed expressed as explosive power [10]. Although release angle is frequently discussed in projectile-based analyses, empirical evidence indicates that release velocity represents the primary determinant of throwing distance, with angle exerting a secondary and velocity-dependent influence [11,12]. In youth athletes, technical inconsistencies may lead to compensatory adjustments in release angle when release velocity is not yet fully optimized. Therefore, performance changes should be interpreted within the broader interaction between kinematic sequencing and velocity generation.

During adolescence, the preparation of junior athletes must balance physical development with sport-specific technical refinement. Previous research on youth javelin throwers has shown that technical inconsistencies during the delivery phase are common, including excessive elbow flexion and large knee flexion of the braking leg, which may reduce the efficiency of force transfer during release [13]. These findings highlight the importance of monitoring technique development during the formative stages of athlete preparation. However, evidence-based biomechanical monitoring during this developmental stage remains limited, particularly in field-based training contexts.

Javelin throwing is a highly demanding discipline characterized by complex three-dimensional biomechanical requirements and substantial neuromuscular stress. Performance outcomes depend on the efficiency of the neuromuscular system and the coordination of the kinetic chain, which governs the sequential transfer of force from the lower to the upper body [14,15]. The release phase is especially critical, as throwing distance is determined by the interaction between release velocity, release angle, and aerodynamic factors. Biomechanical studies consistently report optimal release angles in elite javelin throwers within approximately 32–36°, although this range is influenced by release velocity and aerodynamic conditions [16,17]. In addition, biomechanical analyses of elite female javelin throwers have shown that throwing distance is strongly associated with release velocity and delivery-phase kinematics, including the knee angle of the braking leg and elbow configuration during release [18].

Adaptation in javelin throwing occurs progressively due to the complexity of the movement pattern and the physiological demands of the event. Research has shown that neuromuscular adaptations, including changes in motor unit recruitment and rate of force development, are influenced by training stimuli and athlete-specific factors [19]. Integrated neuromuscular training approaches have been proposed as effective strategies for improving performance by combining strength, speed, coordination, and plyometric components [20].

During adolescence (15–18 years), athletes undergo significant morphological and neuromuscular development, which influences coordination, motor control, and force production capabilities. According to contemporary youth physical development models, adolescence represents a critical window for neuromuscular adaptation, coordination refinement, and force production development [21].

From a motor control perspective, skilled performance is characterized not by rigid repetition but by functional movement variability, allowing adaptable coordination patterns under changing task constraints [22,23]. In complex throwing tasks, controlled variability within the proximal-to-distal sequence may enhance movement stability while preserving performance output.

In throwing events, explosive power represents the dominant biomotor quality, emerging from the interaction of strength and speed capacities. However, increases in general strength do not automatically translate into sport-specific performance improvements. The transfer of training adaptations depends on biomechanical similarity, contraction velocity, and coordination specificity [24,25]. In javelin throwing, effective performance requires precise intersegmental timing and efficient proximal-to-distal force transmission to maximize release velocity.

The high mechanical loads experienced by the musculoskeletal system necessitate careful training design to support adaptation while minimizing injury risk. Achieving advanced motor skill refinement requires precise intersegmental coordination and controlled variability during the release phase. High angular velocities and joint torques during the acceleration phase increase stress on the shoulder and elbow complex, reinforcing the need for technically efficient movement patterns to reduce cumulative overload [15,26].

Despite extensive biomechanical research on elite javelin throwers using laboratory-based three-dimensional motion capture systems [12], limited evidence exists regarding biomechanical characteristics in youth populations assessed under field conditions. Previous technique assessments in junior throwers have identified common mechanical errors during the delivery phase that may influence release efficiency and throwing performance [13]. However, few studies have examined how structured biomechanically oriented training interventions influence coordination-related parameters and release mechanics during adolescence using practical instrumentation applicable to real-world training environments [7,8]. 

The aim of this study was to investigate the functional biomechanical characteristics of javelin throw technique in junior athletes and to evaluate the changes associated with a structured biomechanical training intervention on technique-related performance variables. To our knowledge, no previous study has systematically combined longitudinal biomechanical monitoring with a structured field-based intervention in junior javelin throwers within ecologically valid training settings.

## 2. Materials and Methods

### 2.1. Study Design

This study used a pretest–posttest design involving a single experimental group of junior javelin throwers. A single-group approach was adopted due to the limited availability of performance-level junior javelin throwers within the specified age category at national level. The independent variable was a structured biomechanically oriented training intervention, while dependent variables included throwing velocity, javelin release angle, coordination-related indices (angular velocity and stability parameters), and lower-limb explosive power. Testing was conducted at baseline (January 2025) and post-intervention (April 2025), following a 12-week intervention period.

Participants continued their regular club-based technical training throughout the intervention period. However, no additional structured strength or biomechanically oriented programs outside the experimental protocol were introduced. Training volume and content were coordinated with the coaching staff to ensure consistency across the intervention period.

### 2.2. Participants and Ethical Approval

Fifteen female junior athletes (15–18 years), actively competing at national level, were included in the study. Participants had a mean body mass of 67.6 ± 8.5 kg and a mean height of 166.8 ± 5.0 cm. The athletes had approximately 4–5 years of systematic training experience and trained on average 4–5 sessions per week. The mean personal best performance in the javelin throw was 42.13 ± 5.2 m. All participants were actively involved in national-level competitions in the javelin throw.

Inclusion criteria were as follows: (i) age between 15 and 18 years; (ii) active participation in national-level competition during the current season; (iii) minimum training frequency of five sessions per week for at least six consecutive months prior to baseline testing; and (iv) absence of musculoskeletal injury within the previous three months. Exclusion criteria included the following: (i) any injury occurring during the intervention period; (ii) absence from more than 10% of training sessions; and (iii) incomplete test data at either time point. The study protocol, participant information sheets, and consent forms were submitted to the Research Ethics Committee of Transilvania University of Brașov prior to recruitment and baseline testing. According to the university’s administrative procedure, the committee issues the written approval certificate after completion of data collection and verification of the full study documentation. Written informed consent was obtained from all participants and from parents/legal guardians prior to participation and baseline testing. All procedures were conducted in accordance with the Declaration of Helsinki.

All 15 participants completed both pre-intervention (T1) and post-intervention (T2) assessments. No missing data were recorded, and all analyses were conducted using complete paired datasets (*n* = 15 for all variables).

### 2.3. Setting and Timeline

The research was conducted between October 2024 and June 2025. Testing sessions were performed at the Transilvania University of Brașov, Faculty of Physical Education and Mountain Sports, and at the Iolanda Balaș Soter Stadium in Bucharest, with institutional approvals. The intervention mesocycle was implemented between February and April 2025, and data processing and statistical analysis were completed in May 2025.

### 2.4. Training Intervention

The experimental program was planned over 12 weeks and implemented as three training mesocycles. Each mesocycle included four weekly microcycles, and each microcycle comprised five to six training sessions (total: 67 training plans). The intervention targeted: (i) development of speed and explosive strength (power), (ii) improvement of balance and coordination capacities relevant to throwing, and (iii) optimization of biomechanical and kinematic transfer from the lower to the upper body during the javelin throw. Program content emphasized technique-oriented exercises designed to improve the impulse/momentum phase, intersegmental coordination, and movement efficiency in the approach, impulse stride, and release phases.

The intervention combined technical throwing practice, strength–power training, and coordination drills. Weekly throwing volume ranged between approximately 60–100 submaximal and maximal throws, depending on the mesocycle phase. Throwing intensity progressed from moderate technical emphasis in Mesocycle 1 toward higher-intensity, competition-specific efforts in Mesocycle 3.

Strength training sessions included lower-limb and core exercises performed at moderate-to-high intensities (approximately 60–85% of estimated 1RM), with 3–5 sets of 4–8 repetitions depending on the objective (power or maximal strength). Plyometric exercises were performed in 3–4 sets of 6–10 repetitions with controlled recovery intervals.

Training load progression followed a periodized structure, with gradual increases in intensity and technical specificity across the three mesocycles, while overall weekly volume was adjusted to avoid excessive fatigue. Internal load was monitored qualitatively through coach observation and perceived exertion, ensuring appropriate recovery between sessions. Weekly training load was monitored through session frequency, estimated external load parameters (number of throws, sets, repetitions, relative intensity), and qualitative internal load assessment based on rate of perceived exertion and coach observation. No abrupt changes in overall training volume were introduced during the study period. Representative examples of exercises included: (i) impulse-stride drills emphasizing trunk–pelvis separation timing; (ii) medicine ball rotational throws (2–4 kg) targeting proximal-to-distal sequencing; (iii) plyometric lower-limb exercises (drop jumps, bounding drills) to enhance rate of force development; and (iv) resisted throwing drills with elastic bands to improve arm acceleration mechanics.

Progression across the three mesocycles involved a gradual increase in technical specificity and movement velocity, while overall throwing volume remained within controlled limits (60–100 throws/week), with a shift from submaximal technical throws in Mesocycle 1 to higher-intensity competitive simulations in Mesocycle 3 (Table 1).

### 2.5. Testing Protocol and Outcome Measures

All tests were preceded by a standardized warm-up and were performed under similar environmental conditions. For each test, multiple trials were conducted, and the best performance or the mean value, depending on the test, was retained for statistical analysis. Three categories of assessments were used: motor potential, coordination capacity, and technical performance. All tests were administered at pre- and post-intervention under standardized conditions.

The Gyko inertial measurement unit (Gyko IMU; Microgate, Bolzano, Italy) was used to quantify coordination-related kinematic variables during the coordination capacity tests. The device integrates triaxial accelerometers and gyroscopes to record linear acceleration and angular velocity of body segments. From these signals, movement length (cumulative sensor displacement), movement distance, and movement speed were derived during task execution. Previous studies have reported acceptable validity and reliability of inertial measurement systems for dynamic sport-specific movements when compared with laboratory-based motion capture systems, with intraclass correlation coefficients ranging from 0.80 to 0.95 in functional movement assessments [7,8].

Ball throwing velocity was measured using a radar gun (SpeedTrac X Radar Gun, Bushnell Performance Optics, Overland Park, KS, USA), which determines projectile speed using Doppler radar technology. The manufacturer reports measurement accuracy within ±0.1 m/s for projectile speed assessment.

Two-dimensional kinematic analysis of javelin release angle was performed using Kinovea software (Kinovea v0.9.5; Kinovea Open Source Project, Bordeaux, France). The software allows frame-by-frame video analysis and manual digitization of angular variables during the release phase. Previous studies have demonstrated acceptable validity and reliability of two-dimensional video-based kinematic analysis for angular measurements, including Kinovea-based assessments, when compared with reference methods, although measurement error may vary depending on camera alignment, calibration procedures, and digitization accuracy [9].

For maximal performance variables (ball throwing speed, throwing distance, and drop jump), the best trial was retained for statistical analysis. In contrast, for coordination variables derived from IMU recordings, mean values across three trials were calculated to reduce within-subject variability and improve measurement reliability, as recommended in biomechanical and performance testing protocols [27,28]. This approach was adopted to ensure that performance outcomes reflected maximal execution, whereas coordination outcomes captured more stable movement behavior across repeated trials.

To enhance methodological rigor, all devices were calibrated prior to testing according to manufacturer guidelines. Previous validation studies report acceptable concurrent validity for the Gyko IMU when compared to laboratory-based motion capture systems, with intraclass correlation coefficients ranging between 0.80 and 0.95. The OptoJump system has demonstrated high reliability for jump assessment (ICC > 0.90), and radar-based velocity measurement devices report minimal measurement error (±0.1 m/s). These characteristics support the reproducibility and reliability of the applied field-based instrumentation.

#### 2.5.1. Motor Potential Test Procedures

**Figure 1:**
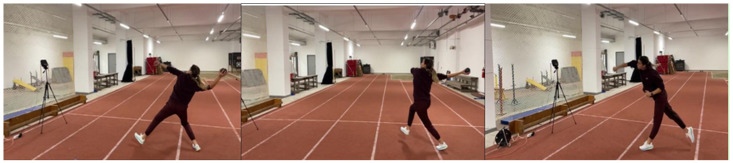
Ball throwing speed assessment performed from a cross-step.

Ball throwing speed: Athletes performed a small-ball throw from a cross-step toward a wall positioned in front of the throwing direction. Ball speed was measured using a SpeedTrac X radar gun (Bushnell Performance Optics, Overland Park, KS, USA). The radar gun was positioned on a tripod directly in front of the athletes, aligned with the direction of the throw to capture the release velocity of the ball. The device was placed approximately 3–4 m from the throwing line at approximately shoulder height and oriented parallel to the trajectory of the ball to minimize angular measurement error. The test was performed using a standard oina ball (mass: 150 g; circumference approximately 22 cm), with identical dimensions used across all trials to ensure consistency of measurement (Figure 1).

**Figure 2:**
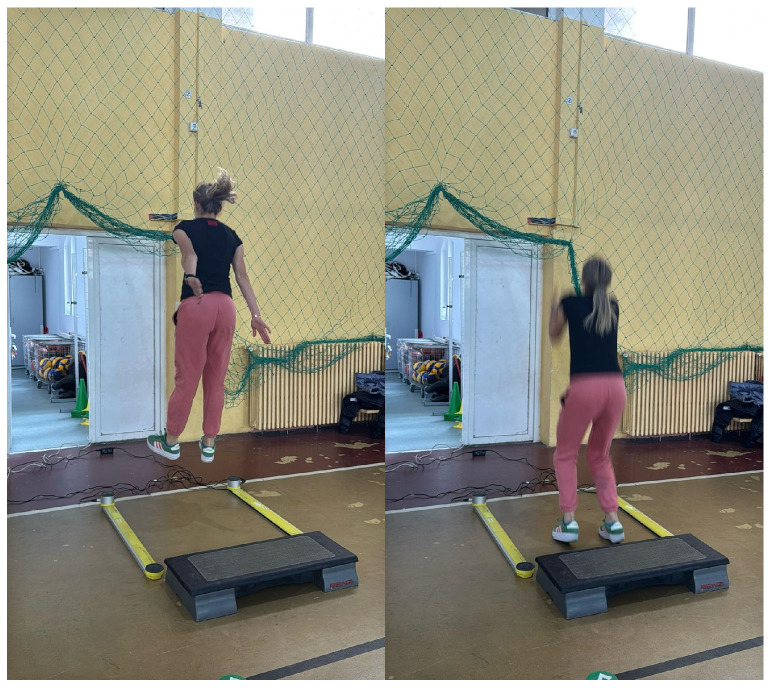
Execution of the drop jump test.

Drop jump: Participants stepped down from a 14 cm platform, landed bilaterally, and immediately performed a reactive vertical jump (Figure 2). Jump performance was recorded using the OptoJump system (version 1.13; Microgate, Bolzano, Italy). The following jump metrics were obtained: reactive strength index (RSI), calculated as flight time divided by contact time, and lower-limb power output expressed in W/kg as estimated by the OptoJump software. These variables were used as indicators of stretch–shortening cycle efficiency and explosive lower-limb performance.

#### 2.5.2. Coordination Capacity Test Procedures

**Figure 3:**
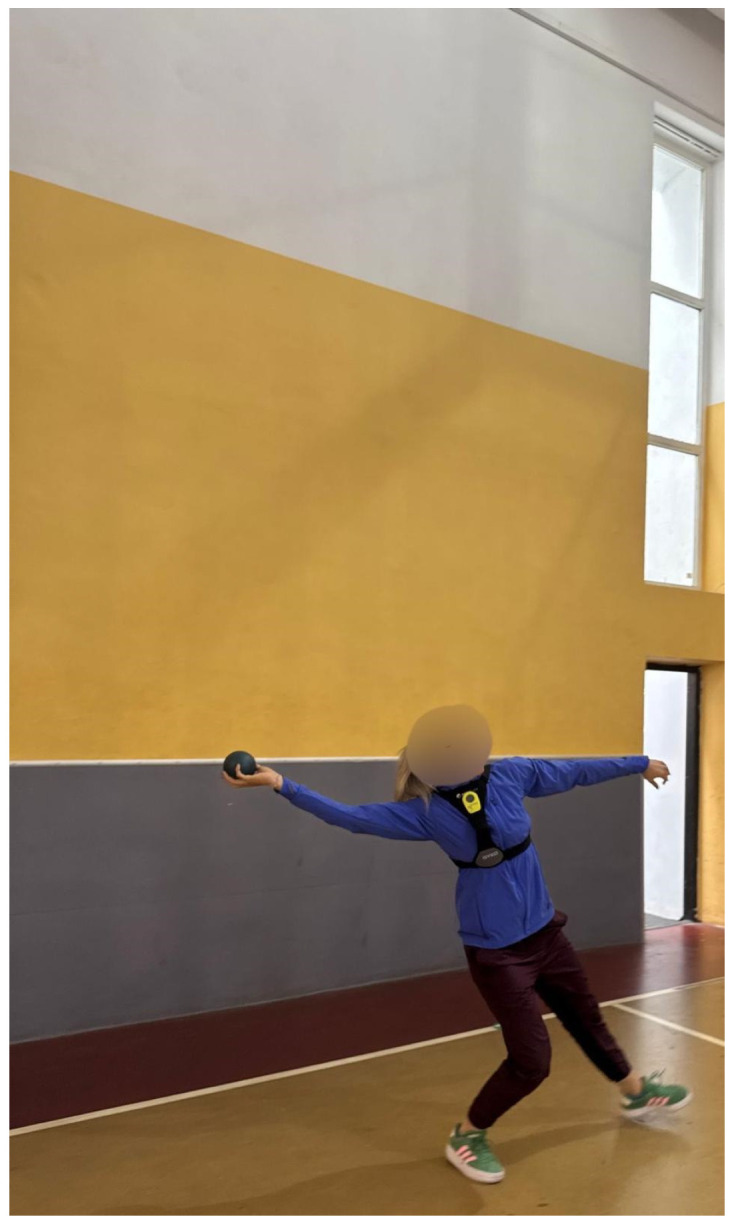
Execution of the entry into the throwing arch test.

Coordination was assessed using two sport-specific tests: entry into the throwing arch and throwing arm balance. These tests were instrumented with a Gyko inertial measurement unit (GYK002; Microgate, Bolzano, Italy; software version 1.1.2.1), which was used to quantify coordination-related parameters. Gyko was positioned on the torso for the throwing-arch entry test and on the wrist for the throwing-arm balance test, to record angular velocity and stability-related parameters. Devices were used according to standardized/validated operational procedures. Entry into the throwing arch: From a bilateral stance, athletes rotated the trunk into the specific throwing-arch position while the sensor was placed on the anterior torso (Figure 3). Each execution lasted approximately 3–4 s, from movement initiation to the final stabilized throwing-arch position.

**Figure 4:**
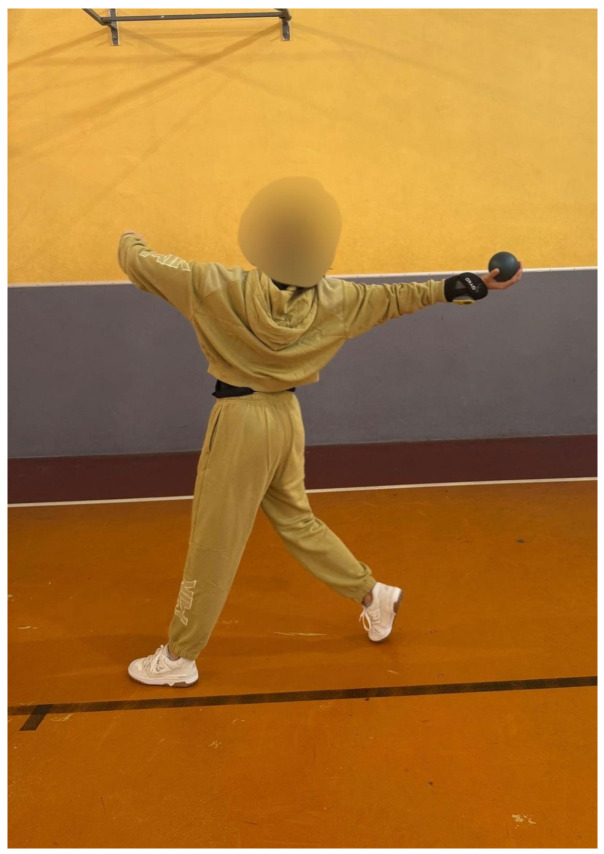
Execution of the throwing arm balance test.

Throwing arm balance: Athletes performed a cross-step with the throwing arm extended backward; the sensor was placed on the wrist to quantify stability and balance of the throwing arm (Figure 4). Each trial lasted approximately 4–5 s, covering the execution of the cross-step and stabilization of the arm position.

##### Signal Processing and Operational Definition of Variables

The Gyko inertial measurement unit integrates a triaxial accelerometer and gyroscope to quantify linear acceleration and angular velocity during movement execution. Data were collected using the manufacturer’s proprietary software (version 1.1.2.1; Microgate, Bolzano, Italy). Signal acquisition was performed at the manufacturer’s default sampling frequency, (100 Hz), and internal filtering algorithms embedded in the proprietary software were applied to reduce high-frequency noise and motion artifacts. No additional external smoothing or filtering procedures were applied beyond those implemented by the manufacturer’s processing pipeline.

For the coordination tests (entry into the throwing arch and throwing arm balance), the following operational variables were extracted:

- Movement length (cumulative sensor displacement) (mm): total cumulative displacement of the sensor during task execution, representing the overall trajectory amplitude relative to the initial reference position.
- Movement distance (mm): maximal linear deviation from the starting position during movement performance.
- Movement speed (cm/s): peak resultant linear velocity derived from accelerometer-based kinematic estimation within the execution phase.
- Stability parameters: variability of angular velocity (expressed as the magnitude of fluctuations in gyroscope-derived angular velocity signals, deg/s), representing trunk or arm oscillation control during task execution.

Anatomical reference frames were defined according to sensor placement (anterior trunk surface for throwing-arch analysis and dorsal wrist for throwing-arm balance assessment). Movements were analyzed within the functional plane of execution corresponding to the specific task.

Prior to each testing session, the sensor was positioned firmly using an elastic strap to minimize relative motion between the device and the body segment. Sensor alignment was standardized according to anatomical landmarks (anterior trunk surface aligned with the sternum for trunk analysis; dorsal wrist aligned with the longitudinal axis of the forearm for arm assessment). A static calibration procedure was performed before testing to define the initial reference orientation.

Although inertial measurement units may be affected by soft-tissue artifact and integration drift during high-velocity movements, the standardized placement and calibration procedures applied were intended to minimize potential sources of variability. In addition, all coordination variables were extracted over the active execution phase defined from movement onset (angular velocity exceeding 5°/s threshold) to task completion, with peak and cumulative values automatically computed using manufacturer algorithms and exported for statistical analysis.

#### 2.5.3. Technical Performance Test Procedures

Javelin release angle: Two-dimensional kinematic analysis was performed using Kinovea software (version 0.9.5; Kinovea Open Source Project, Bordeaux, France) to determine the javelin release angle. The camera was positioned perpendicular to the sagittal plane of motion at a distance of approximately 10–12 m from the release zone and mounted on a fixed tripod at shoulder height to minimize parallax error. Video recordings were obtained at 60 frames per second. Calibration was performed using a 2 m reference object placed within the plane of motion.

The release angle was defined as the angle between the longitudinal axis of the javelin and the horizontal reference line at the instant of release. Frame-by-frame analysis was performed to identify the exact frame corresponding to the moment the javelin left the athlete’s hand, after which the angular measurement was obtained using the Kinovea angle tool.

**Figure 5:**
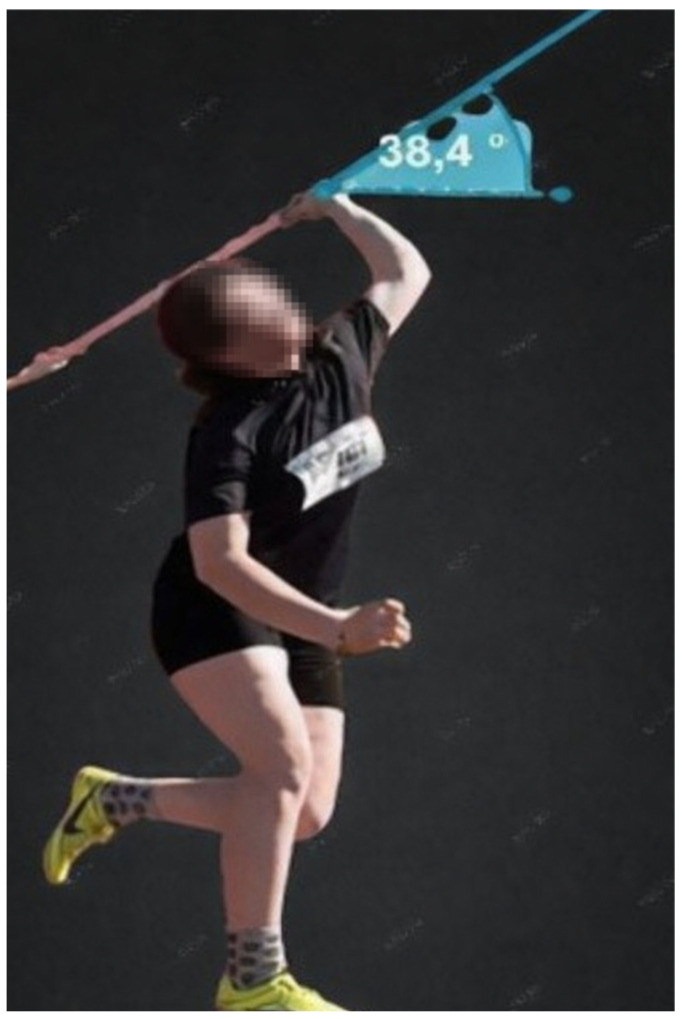
Javelin launch angle.

Throwing distance: Throw distance was measured using a Trimble S5 total station (Trimble Inc., Sunnyvale, CA, USA), which provides high-precision geodetic measurement for field-based distance assessment. For the javelin throw test, the primary technical variables were release angle and throwing distance (Figure 5).

### 2.6. Statistical Analysis

All variables were tested for normality using the Shapiro–Wilk test. Pre- and post-intervention comparisons were performed using paired-sample *t*-tests with 95% confidence intervals. Pearson correlation analysis was conducted to examine the relationship between release angle and throwing distance at both measurement time points. The level of statistical significance was set at *p* < 0.05. Given that multiple paired comparisons were conducted (*n* = 10), a Bonferroni-adjusted significance threshold was applied (*p* < 0.005). Effect sizes were calculated using Cohen’s d for paired samples and interpreted according to conventional thresholds: small (d = 0.20), medium (d = 0.50), and large (d ≥ 0.80). All statistical analyses were performed using IBM SPSS Statistics (version 29.0; IBM Corp., Armonk, NY, USA). A post hoc power analysis was conducted for the primary outcome (throwing distance) using the observed effect size (Cohen’s d = 2.00), sample size (*n* = 15), and a two-tailed significance level of *p* < 0.05. The achieved statistical power was >0.99.

No a priori power analysis was conducted due to the limited availability of performance-level junior javelin throwers within the specified age category. Consequently, the study should be considered exploratory. Although large observed effect sizes suggest adequate statistical sensitivity for primary outcomes, future research should include prospective sample size calculation and controlled designs to strengthen causal inference.

## 3. Results

All statistical analyses were performed on complete paired data from 15 participants (*n* = 15), with no missing values across outcome measures. Mean differences (ΔX) are reported as T1–T2; therefore, negative ΔX values indicate higher post-intervention (T2) scores. All variables met normality assumptions (Shapiro–Wilk, *p* > 0.05). All paired comparisons remained statistically significant after Bonferroni correction (adjusted threshold *p* < 0.005).

Detailed descriptive statistics (mean ± SD) for all variables at pre- and post-intervention are provided in Supplementary Table S1.

### 3.1. Motor Potential Tests

#### 3.1.1. Ball Throwing Speed

Ball throwing speed increased significantly following the intervention (Table 2). The mean improvement was 1.611 m/s (ΔX = −1.611; *p* < 0.001), corresponding to an approximate 6–8% increase in throwing velocity relative to baseline values. The 95% confidence interval (−2.030 to −1.193) did not cross zero, indicating consistent improvement across participants. The observed effect size was very large (d = 2.19). These results indicate a clear improvement in ball throwing velocity following the training intervention.

#### 3.1.2. Drop Jump

Drop jump performance improved for both power output and Reactive Strength Index (RSI) (Table 3), indicating enhanced stretch–shortening cycle efficiency and improved neuromuscular performance following the intervention.

Power output increased by 11.015 W/kg (*p* < 0.001), representing an approximate 8–10% improvement. The confidence interval (−13.180 to −8.850) suggests stable and homogeneous improvement across the sample. The effect size was very large (d = 2.76).

RSI increased by 0.503 (dimensionless; ΔX = −0.503; *p* < 0.001; d = 2.51), corresponding to an estimated 7–9% improvement. The confidence interval (−0.624 to −0.381) did not include zero, and the effect size was also very large (d = 2.51).

### 3.2. Coordination Capacity Tests

#### 3.2.1. Entry into the Throwing Arch

Significant changes were observed in all coordination-related variables related to entry into the throwing arch (Table 4). Overall, the pattern of results indicates a more controlled and efficient execution of trunk positioning during the task following the intervention period.

Movement length (cumulative sensor displacement) decreased significantly (ΔX = 107.123 mm; *p* < 0.001; d = 1.41), representing an approximate 10–15% reduction relative to baseline. The confidence interval (67.407 to 146.839) indicates a consistent decrease across participants. This reduction reflects lower cumulative sensor displacement during trunk rotation.

Movement distance increased modestly (ΔX = 3.068 mm; *p* = 0.001; d = 1.04), with a confidence interval (1.435 to 4.701) indicating consistent change. Movement speed also increased significantly by 70.601 cm/s (*p* < 0.001; d = 1.39), corresponding to an approximate 8–12% improvement, while the confidence interval (42.972 to 98.229) did not cross zero. Together, these changes suggest enhanced movement efficiency and improved dynamic control during trunk rotation.

#### 3.2.2. Throwing Arm Balance

For the throwing arm balance task (Table 5), movement length (cumulative sensor displacement) decreased significantly (ΔX = 136.327 mm; *p* = 0.002; d = 0.96), indicating reduced cumulative displacement during execution. Movement speed also increased significantly (ΔX = 58.616 cm/s; *p* < 0.001; d = 1.19), corresponding to an estimated 7–10% improvement, while the confidence interval (31.684 to 85.548) suggests consistent change across participants. Together, these changes indicate improved stability and more efficient control of the throwing arm during task execution.

### 3.3. Technical Performance Tests

#### Javelin Throw

In the javelin throw test, both release angle and throwing distance changed significantly following the intervention (Table 6). Release angle increased by 8.767° (*p* = 0.001; d = 1.09), with the confidence interval (4.417 to 13.117) indicating consistent improvement across the sample. Throwing distance improved by 2.969 m (*p* < 0.001; d = 2.00), corresponding to an approximate 6–9% increase relative to baseline performance, while the confidence interval (−3.841 to −2.098) did not cross zero, suggesting stable improvement across athletes.

Pearson correlation analysis was conducted to examine the relationship between javelin release angle and throwing distance at both measurement points (T1 and T2). The results are presented in Table 7 and indicate a negative association between these variables, suggesting that higher release angles were associated with shorter throwing distances.

Minimal detectable change (MDC) values calculated from the standard deviation of the paired differences are presented in Table 8. These values provide an estimate of the smallest change that exceeds the measurement error of the applied testing procedures.

## 4. Discussion

The present study investigated functional biomechanical characteristics of javelin throw technique in junior athletes and examined changes associated with a structured biomechanically oriented training intervention. The findings indicate significant pre–post differences across motor potential, coordination capacity, and technical performance variables. However, given the single-group design, these findings should be interpreted as associative rather than causal, as improvements may also reflect biological maturation, seasonal performance progression, or cumulative regular training exposure typical for adolescent athletes.

A negative association between release angle and throwing distance was observed both before and after the intervention (Table 7). This relationship suggests that excessively high release angles may limit throwing distance, highlighting the importance of optimizing release mechanics in junior athletes.

Although statistically significant, these correlations should be interpreted as moderate in practical terms. Release angle explained only part of the variance in throwing distance, highlighting the multifactorial nature of throwing performance. Previous biomechanical studies have shown that throwing distance is primarily determined by release velocity, while release angle exerts a secondary, velocity-dependent effect on performance [11,12,18].

The increase in release angle observed in this study (≈37° to ≈46°) requires cautious biomechanical interpretation. Classical projectile models and three-dimensional analyses suggest optimal release angles lower than 45°, particularly in javelin throwing, where aerodynamic lift, drag forces, and the predominance of horizontal release velocity components play a critical role. In elite athletes, optimal release angles are commonly reported within the range of approximately 32–36°, depending on release velocity and aerodynamic conditions [11,12,17,18].

These findings are partially consistent with previous biomechanical investigations in javelin throwing. Studies conducted in elite populations have consistently reported optimal release angles between approximately 32° and 36°, depending on release velocity and aerodynamic factors [11,12]. In contrast, research in youth and sub-elite throwers has shown greater variability in release parameters, often reflecting developmental differences in trunk rotational speed, segmental coordination, and technical consistency during the delivery phase [13,18]. The higher post-intervention mean release angle observed in the present study may therefore reflect age-specific technical adaptation rather than convergence toward elite biomechanical profiles. Differences between studies may also be explained by methodological variations, including two-dimensional versus three-dimensional kinematic analysis [12].

The persistence of a negative angle–distance correlation after the intervention suggests that excessively high angles remain suboptimal for maximizing throwing distance. Accordingly, the observed increase in mean release angle likely reflects technical adjustment and increased consistency rather than convergence toward an aerodynamically optimal configuration. Previous studies indicate that performance improvements are more strongly related to gains in release velocity and the efficiency of kinetic chain transfer than to angle modification alone [11,12,29].

The reduced magnitude of the negative correlation at T2 may indicate greater technical consistency and reduced variability in release mechanics, potentially reflecting improved motor control during the final phase of the throw. Beyond release angle, high-level biomechanical analyses in elite javelin throwers emphasize the importance of trunk–pelvis separation, proximal-to-distal sequencing, and optimal timing of peak angular velocities in the shoulder and elbow joints.

Beyond release angle, high-level biomechanical analyses in elite javelin throwers emphasize the importance of trunk–pelvis separation, proximal-to-distal sequencing, and optimal timing of peak angular velocities in the shoulder and elbow joints. Three-dimensional motion capture studies have shown that effective proximal-to-distal force transmission requires coordinated rotational acceleration of the pelvis followed by delayed trunk and upper-limb acceleration, while recent modeling studies have further highlighted the role of segmental energy flow in shaping joint loading and movement efficiency [26,29]. The present study did not directly quantify intersegmental timing. However, improvements in coordination variables may indirectly reflect enhanced sequencing efficiency. Nevertheless, without direct 3D kinematic assessment, such mechanistic interpretation remains speculative.

Several recent investigations in throwing and javelin performance have highlighted the importance of specific biomechanical and training factors in sport technique and outcomes. In youth throwing contexts, anthropometric and motor ability profiles have been shown to correlate strongly with release velocity and throw performance, reinforcing the relevance of kinematic variables such as release speed in junior athletes [30].

These findings are consistent with previous research examining key technique elements in ball throwing tasks, where improvements in arm positioning, elbow alignment, and stabilization of the support leg have been identified as important determinants of throwing performance [31]. In particular, the integration of instructional strategies combined with direct feedback has been shown to significantly improve technique execution in throwing tasks. In this context, the improvements observed in coordination-related variables and throwing performance in the present study may reflect similar mechanisms, where targeted technical training and feedback contributed to more efficient movement patterns. Significant improvements were observed in ball throwing speed and drop jump performance, with very large effect sizes. These findings suggest enhanced rate of force development and improved neuromuscular efficiency. From a functional perspective, such adaptations are relevant because they may support more effective force generation during the final acceleration phase of the throw.

The observed improvements in drop jump performance may indicate enhanced stretch–shortening cycle efficiency and improved rate of force development, both of which are critical for impulse generation during the final acceleration phase of the throw. In javelin biomechanics, rapid proximal-to-distal force transmission from the lower limbs through the trunk to the throwing arm is essential for maximizing release velocity [26,29]. Therefore, improvements in Reactive Strength Index may contribute indirectly to enhanced throwing performance by supporting more effective proximal-to-distal force transmission.

The observed improvements in RSI are consistent with previous literature on plyometric and neuromuscular training in youth athletes. Markovic and Mikulic [20] reported that lower-extremity plyometric training enhances stretch–shortening cycle efficiency and rate of force development, both of which are critical for explosive sports performance. Similarly, neuromuscular training models emphasize the importance of integrating strength and coordination stimuli during adolescence to maximize transfer to sport-specific tasks. Although the present study did not isolate plyometric training effects independently, the magnitude of improvement in drop jump performance aligns with findings from integrated strength–power training interventions reported in youth populations [21].

However, extremely large effect sizes (d > 2.0) should be interpreted cautiously in small samples. Such magnitudes may partly reflect within-subject dependency, low baseline variability, or training-task similarity. While the improvements are practically meaningful, replication with larger samples is required to confirm the magnitude of these effects.

The minimal detectable change (MDC) values provide additional context for interpreting the practical significance of the observed changes. For several variables, including ball throwing speed and drop jump performance, the magnitude of improvement exceeded the corresponding MDC values, suggesting that these changes likely reflect true performance adaptations beyond measurement error. In contrast, for variables such as release angle and throwing distance, the observed mean changes were smaller than the calculated MDC thresholds. This indicates that, despite statistical significance, these changes should be interpreted with caution, as they may fall within the limits of measurement variability. Therefore, MDC analysis highlights the importance of considering both statistical and measurement sensitivity indicators when evaluating training-induced adaptations.

Significant reductions in movement length were observed in both coordination tasks. Mechanically, movement length represented the cumulative sensor displacement during task execution. In the context of these coordination tasks, excessive displacement may reflect compensatory trunk oscillations or reduced segmental control. A reduction in movement length therefore likely indicates more controlled trunk stabilization and reduced extraneous movement during rotational preparation. In javelin throwing, minimizing unnecessary trunk and arm oscillation may support more efficient proximal-to-distal sequencing and improve mechanical energy transfer toward the release phase [14,29]. Thus, reduced movement length may reflect improved coordination efficiency rather than restricted movement amplitude.

Mechanistically, detailed biomechanical modeling of the acceleration phase in javelin throwing underscores the importance of segmental energy transfer and resulting joint torques on movement execution and injury risk, providing a framework for understanding lower-to-upper body coordination improvements observed in this study [29]. In this context, the observed changes in coordination-related variables may reflect more efficient transmission of mechanical energy along the kinetic chain during the throwing action.

Improvements in coordination-related indices may also be interpreted in light of established kinetic chain models in throwing biomechanics. Sequential segmental activation and trunk–pelvis dissociation have been identified as critical determinants of efficient proximal-to-distal force transmission in overarm throwing [14,26]. Although direct intersegmental timing was not quantified in the present study, the reduction in extraneous trunk displacement and increased movement speed are consistent with improved proximal-to-distal sequencing reported in laboratory-based biomechanical analyses. The present findings extend these observations to an ecologically valid, field-based training context in junior athletes.

Nevertheless, because some coordination tests were conceptually related to exercises included in the intervention, a learning or familiarization effect cannot be excluded. Improvements may therefore reflect both neuromuscular adaptation and task-specific practice.

The periodized structure of the three mesocycles likely contributed to the observed adaptations. The initial phase emphasized balanced development of strength–speed and coordination, followed by increased technical specificity and velocity emphasis. Such progression aligns with principles of long-term athletic development and may support neuromuscular consolidation during adolescence.

Although the present study did not employ full three-dimensional motion capture to quantify segmental sequencing directly, the measured variables can be interpreted within established biomechanical models of javelin throwing. Improvements in ball throwing speed, drop jump RSI, and coordination indices may reflect enhanced proximal-to-distal force transmission and more efficient impulse transfer along the kinetic chain. In throwing mechanics, effective trunk–pelvis dissociation and timely angular velocity sequencing are known to facilitate optimal proximal-to-distal force transmission from the lower limbs to the throwing arm. While these variables were not directly quantified using 3D kinematics, the observed changes in coordination stability and Reactive Strength Index are consistent with improved neuromuscular conditions supporting segmental timing efficiency.

From an applied standpoint, the results suggest that integrating strength–power training, coordination drills, and structured technical feedback may support improvements in biomechanical efficiency in junior throwers. However, due to the absence of a control group, these interpretations should be considered cautiously. The observed improvements cannot be attributed exclusively to the intervention, as they may also reflect normal developmental progression, seasonal training adaptations, or accumulated technical practice typical of adolescent athletes.

From a coaching perspective, biomechanically oriented training programs for junior javelin throwers should integrate:

(i) Strength–power development targeting rate of force production;
(ii) Coordination drills emphasizing trunk stabilization and proximal-to-distal sequencing;
(iii) Structured video-based technical feedback to enhance release consistency and segmental timing.

Importantly, coaches should avoid overemphasizing isolated modification of release angle without considering release velocity and intersegmental coordination. Technical optimization should be individualized and embedded within the broader kinetic chain framework.

From a training perspective, the previous literature on javelin training methods reports positive effects of strength, plyometric, and coordination exercises on throw outcomes, supporting the combined approach used in the present intervention [32]. These findings align with our observed increases in explosive power and coordination indices following training.

### Limitations

The single-group pretest–posttest design represents an important methodological limitation. Without the inclusion of a control group, it is not possible to determine the independent contribution of the intervention to the observed changes. Improvements may partially reflect normal developmental processes, seasonal training effects, or cumulative technical practice typical for adolescent athletes. Consequently, the present findings should be interpreted as indicative of potential associations between the training program and performance changes rather than definitive evidence of a causal intervention effect. Future research employing randomized or matched control-group designs is required to isolate the specific contribution of biomechanically oriented training interventions.

In addition, the magnitude of some observed effect sizes, particularly for drop jump power and throwing distance, may appear substantial relative to typical seasonal adaptations. While the structured intervention likely contributed to these changes, normal biological maturation and cumulative training exposure cannot be fully disentangled within the present design. The relatively small sample size (*n* = 15) restricts generalizability and may inflate observed effect sizes, and conceptual similarity between certain coordination tests and training exercises introduces potential learning bias.

Furthermore, the use of wearable inertial sensors and two-dimensional video analysis introduces measurement limitations compared to three-dimensional motion capture systems. The use of two-dimensional video analysis for release angle determination represents a practical field-based solution but does not fully capture the three-dimensional complexity of javelin throwing, and perspective and parallax errors may have influenced angle estimation. In addition, no a priori power analysis was conducted due to limited participant availability; therefore, the study should be considered exploratory, and findings require confirmation through controlled designs with larger samples. Although Bonferroni correction was applied to reduce the risk of Type I error, the relatively small sample size may still limit statistical generalizability, and effect size interpretation should be considered alongside confidence intervals rather than *p*-values alone.

An additional limitation relates to measurement sensitivity. For several variables, the observed changes did not exceed the minimal detectable change (MDC), indicating that some differences may fall within the limits of measurement error. Therefore, caution is required when interpreting small but statistically significant changes, particularly for variables derived from field-based instrumentation.

## 5. Conclusions

Within the limits of a single-group exploratory design, the present findings suggest that the proposed biomechanically oriented training program was associated with improvements in motor potential, coordination capacity, and technical performance in junior javelin throwers. These results should be interpreted cautiously, as the absence of a control group does not allow for causal inference, but they provide preliminary evidence supporting the potential relevance of biomechanically informed training approaches in youth athletes.

In terms of coordination capacity, meaningful progress was identified in both the entry into the throwing arch and throwing arm balance tests. These improvements suggest enhanced trunk stability, intersegmental coordination, and control of the throwing arm during key phases of the throwing motion. The observed reductions in movement length (cumulative sensor displacement), together with increases in movement speed, suggest more stable and efficient execution during these tasks.

Technical performance analysis showed that changes in technique-related indicators were accompanied by improvements in throwing distance. Modifications in javelin release angle and throwing distance further support the effectiveness of integrating biomechanical and coordinative elements within the training process.

The structured integration of strength, speed, coordination, and technical exercises was associated with changes consistent with improved proximal-to-distal force transmission during the throwing action. The use of video-based kinematic analysis and inertial measurement tools facilitated objective assessment of technique-related variables and supported targeted technical refinement. Overall, the results suggest that a functional biomechanical approach to training may support improvements in movement efficiency and performance outcomes in junior javelin throwers.

From an applied perspective, the implementation of the proposed training program was associated with measurable improvements in individual athletic performance. These findings underline the practical relevance of biomechanically informed training interventions and their potential contribution to the long-term development of junior athletes in throwing events.

However, due to the absence of a control group, the results cannot be interpreted as evidence of a direct causal effect of the intervention. Future randomized or controlled longitudinal studies incorporating three-dimensional kinematic analysis are required to confirm the independent contribution of structured biomechanical training to performance development in youth javelin throwers.

## Figures and Tables

**Table 1:** Summary of training themes distribution across mesocycles.

Theme	Objective	Mesocycle 1 Training Sessions	Mesocycle 2 Training Sessions	Mesocycle 3 Training Sessions	Total Number of Sessions
Speed	Developing the reaction speed of the throwing arm Developing the speed of the motion throwing during the last 3 steps Developing the speed of the throwing arm.	3	3	4	10
		3	3	2	8
		3	3	3	9
Power	Developing lower limb strength Developing plyometric strength Developing core strength	4	3	2	9
		4	3	2	9
		4	3	4	11
Coordinative abilities	Developing coordination between throwing and trunk/arm rotation Developing specific balance Developing throwing arm skills	3	4	5	12
		3	4	5	12
		3	4	5	12
Technique	Improving throwing Improving trunk rotation Improving throwing technique	3	4	4	11
		3	4	4	11
		3	4	6	13
Video Analysis	Video meetings with athletes Video meetings with coaches	4	3	4	11
		4	3	4	11
Total sessions per mesocycle		47	48	54	149

Note: The reported number of sessions refers to the frequency of specific training themes within the microcycles and mesocycles. A single training session could include multiple themes (e.g., speed, strength, coordination, or video analysis); therefore, the total counts represent thematic exposures rather than discrete training sessions.

**Table 2:** Descriptive Ball Throw Speed Statistics—differences.

Test	ΔX	SD	*t*	*df*	*d*	*p*	95% CI	
							Lower	Upper
Ball throw speed (m/s) (T1–T2)	−1.611	0.736	−8.474	14	2.19	<0.001	−2.030	−1.193

*n* = 15; ΔX—Mean differences; SD—Standard deviation; *t*—t-student value; *df*—degrees of freedom; *d*—Cohen’s d; *p*—statistical significance threshold; 95% CI—95% confidence interval.

**Table 3:** Descriptive Drop Jump Statistics—differences.

Test	ΔX	SD	*t*	*df*	*d*	*p*	95% CI	
							Lower	Upper
Drop jump—Power (W/kg)	−11.015	3.987	−10.698	14	2.76	<0.001	−13.180	−8.850
Drop jump—Reactive Strength Index (-)	−0.503	0.200	−9.735	14	2.51	<0.001	−0.624	−0.381

*n* = 15; ΔX—Mean differences; SD—Standard deviation; *t*—t-student value; *df*—degrees of freedom; *d*—Cohen’s d; *p*—statistical significance threshold; 95% CI—95% confidence interval.

**Table 4:** Descriptive statistics of the entry into throwing arch—differences.

Test	ΔX	SD	*t*	*df*	*d*	*p*	95% CI	
							Lower	Upper
Entry into throwing arch—Length (mm)	107.123	75.954	5.462	14	1.41	<0.001	67.407	146.839
Entry into throwing arch—Distance (mm)	3.068	2.938	4.045	14	1.04	<0.001	1.435	4.701
Entry into throwing arch—Speed (cm/s)	70.601	50.769	5.386	14	1.39	<0.001	42.972	98.229

*n* = 15; ΔX—Mean differences; SD—Standard deviation; *t*—t-student value; *df*—degrees of freedom; *d*—Cohen’s d; *p*—statistical significance threshold; 95% CI—95% confidence interval.

**Table 5:** Descriptive statistics of the throwing arm balance—differences.

Test	ΔX	SD	*t*	*df*	*d*	*p*	95% CI	
							Lower	Upper
Throwing arm balance—Length (mm)	136.327	141.362	3.735	14	0.96	0.002	54.881	217.772
Throwing arm balance—Speed (cm/s)	58.616	49.364	4.599	14	1.19	<0.001	31.684	85.548

*n* = 15; ΔX—Mean differences; SD—Standard deviation; *t*—t-student value; *df*—degrees of freedom; *d*—Cohen’s d; *p*—statistical significance threshold; 95% CI—95% confidence interval.

**Table 6:** Descriptive statistics of javelin release angle and javelin throw distance—differences.

Test	ΔX	SD	*t*	*df*	*d*	*p*	95% CI	
							Lower	Upper
Javelin release angle (°)	8.767	8.011	4.238	14	1.09	0.001	4.417	13.117
Javelin throw distance (m)	−2.969	1.488	−7.731	14	2.00	<0.001	−3.841	−2.098

*n* = 15; ΔX—Mean differences; SD—Standard deviation; *t*—t-student value; *df*—degrees of freedom; *d*—Cohen’s d; *p*—statistical significance threshold; 95% CI—95% confidence interval.

**Table 7:** Correlation between the angle of javelin release and throwing distance.

		Throwing Distance (m)—T1	Throwing Distance (m)—T2
Release angle (degrees)—T1	Pearson correlation	−0.678	
	*p*	0.005	
	*n*	15	
Release angle (degrees)—T2	Pearson correlation		−0.456
	*p*		0.048
	*n*		15

**Table 8:** Measurement sensitivity indicators for the assessed variables.

Variable	MDC
Ball throw speed (m/s)	2.04
Drop jump power (W/kg)	11.05
Drop jump RSI (-)	0.55
Entry arch length (mm)	210.6
Entry arch distance (mm)	8.15
Entry arch speed (cm/s)	140.7
Arm balance length (mm)	392.1
Arm balance speed (cm/s)	136.7
Release angle (°)	22.2
Throw distance (m)	4.12

## Data Availability

The data that support the findings of this study are available from the corresponding author upon reasonable request.

## Supplementary Materials

The following supporting information can be downloaded at: https://www.mdpi.com/article/10.3390/jfmk11020145/s1. Supplementary Table S1: Descriptive statistics (mean ± SD) for all variables at pre- and post-intervention.
