# Supplementary material for: Functional Biomechanical Analysis of Javelin Throw Technique in Junior Athletes
Source: J Funct Morphol Kinesiol. 2026 Mar 31;11(2):145. doi: 10.3390/jfmk11020145 (PMC13108131; doi:10.3390/jfmk11020145)
Supplement: Supplementary file 1 [file jfmk-11-00145-s001.zip › jfmk-4168822-supplementary.pdf]

Table S1. Descriptive statistics (mean ± SD) for pre- (T1) and post-intervention (T2) measurements.

Variable	T1 (Mean ± SD)	T2 (Mean ± SD)
Ball throw speed (m/s)	8.89 ± 1.19	10.50 ± 1.52
Drop jump power (W/kg)	24.93 ± 3.88	35.95 ± 6.46
Drop jump RSI (-)	0.69 ± 0.17	1.20 ± 0.31
Entry arch length (mm)	583.92 ± 62.08	476.79 ± 39.22
Entry arch distance (mm)	18.75 ± 2.91	15.68 ± 2.68
Entry arch speed (cm/s)	251.34 ± 45.28	180.74 ± 20.63
Arm balance length (mm)	536.50 ± 118.64	400.17 ± 44.28
Arm balance speed (cm/s)	167.91 ± 49.82	109.29 ± 7.92
Release angle (°)	57.07 ± 9.50	48.30 ± 9.60
Throw distance (m)	27.55 ± 4.31	30.52 ± 5.14

*n = 15; values are presented as mean ± standard deviation (SD).
